# Antibacterial activity of bioemulsifiers/biosurfactants produced by *Levilactobacillus brevis* S4 and *Lactiplantibacillus plantarum* S5 and their utilization to enhance the stability of cold emulsions of milk chocolate drinks

**DOI:** 10.1002/fsn3.3740

**Published:** 2023-10-10

**Authors:** Galvany Franck Yamagueu Tchakouani, Hippolyte Tene Mouafo, Richard Marcel Nguimbou, Nadège Donkeng Nganou, Augustin Mbawala

**Affiliations:** ^1^ Department of Food Sciences and Nutrition, National School of Agro‐Industrial Sciences University of Ngaoundéré Ngaoundéré Cameroon; ^2^ Centre for Food, Food Security and Nutrition Research Institute of Medical Research and Medicinal Plant Studies Yaoundé Cameroon; ^3^ Department of Food Engineering and Quality Control University Institute of Technology, University of Ngaoundéré Ngaoundéré Cameroon

**Keywords:** antimicrobial activity, bioemulsifiers/biosurfactants, chocolate milk drink, *Lactiplantibacillus plantarum* S5, *Levilactobacillus brevis* S4, *pendidam*, stabilizer

## Abstract

Chocolate milk drink, one of the most popular and widely consumed milk products among the population, independent of their age, has as its main challenge the problem of its physical instability. The aim of this study was to assess the stabilizing effect of bioemulsifiers/biosurfactants (BE/BS) from two lactobacilli strains in a cold chocolate milk drink. The strains *Levilactobacillus brevis* S4 and *Lactiplantibacillus plantarum* S5 isolated from *pendidam* were screened for their ability to produce BE/BS. The produced BE/BS were characterized, their antimicrobial activities were assessed, and their ability to stabilize cold chocolate milk drinks was determined. The results obtained showed BE/BS yields of 3.48 and 4.37 g/L from *L. brevis* S4 and *L. plantarum* S5, respectively. These BE/BS showed emulsifying and surface activities that remained stable after treatment at different temperatures, pH, and salinity. The emulsions formed using BE/BS were stable for 72 h at room temperature (25 ± 1°C). The BE/BS exhibited antimicrobial activity against *Staphylococcus aureus* S1 and *Escherichia coli* E1. When applied to cold chocolate milk drinks at 0.2% (w/v), the BE/BS from *L. brevis* S4 and *L. plantarum* S5 showed interesting solubility indexes and water absorption capacities, which led to the successful stabilization of the drinks. The results of this study demonstrate the stabilizer potential of BE/BS from *L. brevis* S4 and *L. plantarum* S5 and suggest their use in the dairy and food industries.

## Graphical abstract

The issue of the physical instability of chocolate milk drinks, a popular and widely consumed milk product among the population independent of their age, has led to the search for stabilizers. The green alternative proposed in this study was the bioemulsifiers/biosurfactants (BE/BS) from *Levilactobacillus brevis* S4 and *Lactiplantibacillus plantarum* S5. These biomolecules demonstrated good in vitro emulsifying, emulsion stabilizing and antimicrobial activities. Thanks to their interesting solubility indexes and water absorption capacities, the BE/BS have successfully stabilized the cold chocolate milk drinks, thus suggesting their potential use in the dairy industry as well as the food industries as a substitute for chemical stabilizers.

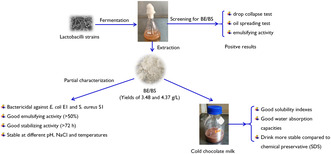

## 1 INTRODUCTION

Chocolate milk can be defined as a complex multiphase that contains fine solid particles from ingredients used for its preparation, such as cocoa, sugar, and certain milk components in a continuous fat phase made of milk fat, cocoa butter, and an emulsifier (Saidin et al., 2014). It is one of the most popular, accepted, and consumed milk products worldwide (Prakash et al., 2010; Thompson et al., 2004). Chocolate milk is incorporated into the modern lifestyle of several populations (Hardiyanti et al., 2020). However, one of the main problems associated with this drink is its physical instability, owing to the sedimentation of cocoa particles (Prakash et al., 2010). Hence, preventing this physical instability that leads to the modification of the sensory attributes of chocolate milk has become nowadays a major technological and scientific challenge for the dairy industry (Aliakbarian et al., 2017; Sher et al., 2013). To solve this problem, stabilizers have been highlighted as the main alternatives (Tasneem et al., 2014). Some stabilizers such as carrageenan, soya lecithin, starch, pectin, xanthan gum, and alginate have been used (Eze et al., 2021; Gama et al., 2019). As consumers' health concerns are growing worldwide, research is directed towards biosurfactants that have potential health‐promoting effects such as antimicrobial activity, antioxidant activity, and immune‐boosting properties. Therefore, biosurfactants appear to be good alternatives.

Biosurfactants are amphiphilic compounds derived from bacteria, yeasts, and molds that possess hydrophobic (hydrocarbon chains) and hydrophilic (carbohydrates, alcohols, ethers, peptides, amino acids, phosphates, or carboxyl acids) moieties that can interact with surfaces and reduce interfacial and surface tensions (Mouafo et al., 2022; Santos et al., 2016). Biosurfactants are of interest because of their green production, low toxicity, structural diversity, high selectivity, greater biodegradability, and stability over a wide range of temperatures, pHs, and salinities (De et al., 2020; Mouafo et al., 2022; Nitschke & Silva, 2018; Silva et al., 2020). The emulsifying activities as well as the emulsions' stabilizing capacity of biosurfactants have been demonstrated in the literature (Mouafo et al., 2022). In addition, biosurfactants have several activities such as antimicrobial, antioxidant, antiadhesive, and immunomodulatory activities (Merghni et al., 2017; Morais et al., 2017; Mouafo, Mbawala, et al., 2020), which might be useful in fighting and/or preventing foodborne diseases associated with pathogens as well as cardiometabolic diseases (Kumar et al., 2021).

The emulsifying and stabilizing properties of biosurfactants have been successfully explored in various food products (Campos et al., 2015, 2019; Farheen et al., 2016; Mbawala et al., 2015; Pessôa et al., 2019; Ribeiro et al., 2020; Silva et al., 2020). To our knowledge, the emulsifying and stabilizing properties of microbial biosurfactants have not yet been tested in chocolate milk drinks. In this context, where there is a need for novel biosurfactants with potential food applications, the present study was designed. Our interest was directed towards biosurfactants from lactobacilli because of their GRAS status. These bacteria produce biosurfactants as a complex mixture of several components, including polysaccharides, lipids, proteins, and phosphates (Ferreira et al., 2017; Mouafo et al., 2022; Mouafo, Mbawala, & Ndjouenkeu, 2018; Mouafo, Mbawala, Tchougang, et al., 2018). Their biosurfactants have demonstrated a good ability to form and stabilize emulsions (Mbawala et al., 2015; Mouafo, Mbawala, & Ndjouenkeu, 2018; Sharma et al., 2016). The general objective of this study was to assess the stabilizing effects of bioemulsifiers/biosurfactants (BE/BS) from *Levilactobacillus brevis* S4 and *Lactiplantibacillus plantarum* S5 in cold chocolate milk drinks.

## 2 MATERIALS AND METHODS

### 2.1 Microorganisms and culture conditions

*Levilactobacillus brevis* S4 and *Lactiplantibacillus plantarum* S5 isolated from an indigenous fermented milk (*pendidam*) were obtained from the culture collection of the Laboratory of Food Microbiology and Biotechnology of the National School of Agro‐industrial Sciences, University of Ngaoundéré, Cameroon. From the frozen stocks stored at −21°C in MRS broth (LiofilChem) containing 20% (v/v) glycerol (BDH Laboratory Supplies), the strains were sub‐cultured twice in 10 mL of MRS broth at 37°C for 24 h. They were then streaked on MRS agar (LiofilChem), and the plates were incubated for 24 h at 37°C. Colonies from these plates were used in the experiments.

Strains of *E. coli* E1 and *S. aureus* S1 were obtained from the culture collection of the Laboratory of Food Microbiology and Biotechnology of the National School of Agro‐industrial Sciences, University of Ngaoundéré, Cameroon. They were inoculated in 10 mL of Trypticase Soya Broth (TSB, LiofilChem) and cultured at 37°C for 24 h. Thereafter, they were streaked on Mueller Hinton agar (MH, LiofilChem) and incubated at 37°C for 24 h. Five well‐isolated colonies that appeared on Petri dishes after incubation were suspended in 5 mL of sterile saline, serially diluted, counted, and adjusted to 8 log CFU/mL.

### 2.2 Screening of the production of bioemulsifiers/biosurfactants

The ability of *L. brevis* S4 and *L. plantarum* S5 to produce BE/BS was assessed using the oil spreading test and emulsification index following the protocols reported by Fookao et al. (2022). A colony from an overnight subculture of the lactobacilli strains on MRS agar was introduced into 15 mL of sterile MRS broth free of tween 80. The tubes were then incubated for 72 h at 37°C under static conditions. After 72 h, the supernatants were collected by centrifugation (4000×*g*, 15 min, 4°C). For the oil spreading test, 20 mL of sterile distilled water was introduced into a Petri dish and 20 μL of refined palm oil (Mayor Oil brand) was carefully deposited. Once the oil deposited on the distilled water spread and formed a thin film, 20 μL of the supernatant was carefully deposited at the center of the oily film. After 1 min, the diameter of the clear zone appearing in the oily film was measured and subtracted from that recorded with MRS broth free of tween 80. Sodium dodecyl sulfate (SDS) (Merck, France) at a concentration of 1% (w/v) was used as a positive control, while distilled water was used as a negative control.

Regarding the emulsification index, 1.5 mL of the supernatant was mixed with 1.5 mL of refined palm oil. The mixture was vortexed (2 min) and left on the bench at room temperature (25 ± 1°C) for 24 h. The height of the emulsion layer formed in the tube and the total height of the liquid in the tube were measured and used to calculate the emulsification index using the following formula:

$$E_{24}=\frac{\text{Height of the emulsion layer}\;\left(\mathrm{mm}\right)}{\text{Total height of the liquid in the tube}\;\left(\mathrm{mm}\right)}\times 100$$

SDS 1% (w/v) and distilled water were used as the positive and negative controls, respectively.

### 2.3 Production of bioemulsifiers/biosurfactants

BE/BS were produced following the method described by Mouafo, Mbawala, and Ndjouenkeu (2018) with slight modifications. In this protocol, 1 mL of an overnight subculture of the lactobacilli strain was introduced into a tube containing 15 mL of sterile MRS broth free of tween 80 and cultured at 37°C for 18 h. The contents of the tube were transferred into 600 mL of sterile MRS broth free of tween 80, followed by incubation under static conditions at 37°C for 72 h. After incubation, the broth was centrifuged (4000×*g*, 15 min, 4°C), the bottom was discarded, and the supernatant was collected. BS/BS was extracted from the supernatant using the ethanol precipitation method (Fookao et al., 2022; Gnanamani et al., 2010). Briefly, the supernatant was mixed with an equal volume of chilled ethanol, and the mixture was incubated at 4°C for 16 h. The precipitate obtained after incubation was collected by centrifugation (4000×*g*, 15 min, 4°C), washed twice with sterile distilled water, lyophilized, and weighed.

### 2.4 Emulsifying and surface activities of crude bioemulsifiers/biosurfactants

The emulsifying activity of crude BE/BS was determined according to the method described by Fookao et al. (2022). Briefly, 1.5 mL of crude BE/BS at 40 mg/mL in sterile distilled water was mixed with refined palm oil (1.5 mL), and the mixture was vortexed for 2 min before being incubated for 24 h at room temperature (25 ± 1°C). The emulsification index (*E*_24_) was calculated using the formula described above. The emulsions' stability was assessed after 24, 48, and 72 h of incubation at room temperature (25 ± 1°C). The surface activity of crude BE/BS was assessed using the oil spreading test as previously described by Fookao et al. (2022). Twenty microliters of a solution of BE/BS (40 mg/mL) were deposited on the surface of a thin oily film, and the diameter of the clearing zone that appeared after 1 min was measured.

For both tests, SDS 1% (w/v) and distilled water were used as the positive and negative controls, respectively.

### 2.5 Stability studies of crude bioemulsifiers/biosurfactants

The stability of emulsifying and surface activities of crude BE/BS was assessed at different temperatures, NaCl concentrations, and pH values. For thermal stability, crude BE/BS solutions were prepared at 40 mg/mL and heated at 25, 50, and 100°C for 15 min, and their emulsification index and surface activities were measured as described above. The solutions of BE/BS were also autoclaved (121°C, 1.5 bar, 15 min), allowed to cool at room temperature (25 ± 1°C), and their surface and emulsifying activities were determined. With regard to pH stability, the pH of the solutions of crude BE/BS was adjusted to 4, 6, 8, and 10 using HCl 1 N (Merck) or NaOH 1 N (Merck) solutions, and their emulsification index and surface activities were measured as described above. Regarding the stability of salinity, the NaCl (Merck) concentrations of crude BE/BS solutions prepared at 40 mg/mL were adjusted to 0, 25, and 50 g/100 mL, and their emulsification index and surface activities were measured as described above.

### 2.6 Antibacterial activity of crude bioemulsifiers/biosurfactants

#### 2.6.1 Qualitative assessment

The qualitative antibacterial activity of crude BE/BS was assessed using the disk diffusion method of the American Society for Microbiology (Cavalieri et al., 2005) with slight modifications. Under aseptic conditions, 100 μL of each pathogen suspension (8 log CFU/mL) was surface‐inoculated into Petri dishes containing sterile MH agar. Petri dishes were allowed to dry on the bench at room temperature (25 ± 1°C) for 1 h. Sterile disks (diameter 6 mm) were then deposited on the surface of the inoculated MH agar, and 20 μL of crude BE/BS solution at 40 mg/mL was added to the disks. Petri dishes were incubated at 37°C for 24 h, and the diameter of the inhibition zone was measured. Gentamicin (Titan Biotech Ltd.) and ampicillin (Titan Biotech Ltd.) were used as controls.

#### 2.6.2 Quantitative assessment

##### Determination of minimum inhibitory concentration (MIC)

The MIC of different crude BE/BS was determined using the macro dilution method of the American Society for Microbiology (Cavalieri et al., 2005) with slight modifications. Under aseptic conditions, 4 mL of sterile trypticase soy broth (TSB, LiofilChem) was introduced into the test tubes. Then, 0.5 mL of an overnight culture of the pathogen suspension adjusted to 8 log CFU/mL was added. An aliquot (0.2 mL) of the crude BE/BS solution was introduced into the tubes, and their final concentrations (0, 12.5, 25, 50, 75, and 100 mg/mL) were adjusted using sterile TSB. After homogenization, the tubes were incubated for 24 h at 37°C. A test tube prepared under the same conditions and free of pathogens and antimicrobials was used as the growth control. After 24 h of incubation, the tube containing the lowest concentration of crude BE/BS, where no bacterial growth was observed by the naked eye, was considered as the MIC. To confirm MIC readings, 40 μL of a solution of 2,3,5 triphenyltetrazolium chloride (Riedel‐deHaën Ag Seelze‐Hannover) at 0.2 mg/mL was added into test tubes, followed by incubation at 37°C for 2 h. The color change from red to purple indicated the presence of microorganisms, whereas no change in color indicated the complete inhibition of microorganisms present in the test tubes (Chebaibi et al., 2016).

##### Determination of minimum bactericidal concentration (MBC)

The method of the American Society for Microbiology (Cavalieri et al., 2005), with slight modifications, was used to determine the MBC of the different crude BE/BS. One hundred microliters were taken in tubes from MIC determination, into which no growth was observed, and surface inoculated onto sterile MH agar. The Petri dishes were incubated at 37°C for 24 h, and the lowest concentration of crude BE/BS for which no growth was observed after incubation was considered as the MBC.

### 2.7 Application of crude BE/BS to the stabilization of a cold chocolate milk drink

The cold chocolate milk drink was prepared by dissolving 1 g of chocolate milk powder (Chococam brand) in 10 mL of sterile distilled water. The mixture was homogenized and stored at 4°C. In this study, crude BE/BS at different concentrations (0%, 0.2%, 0.3%, and 0.5%: w/v) were combined with 1 g of chocolate milk powder in 10 mL of sterile distilled water. The choice of the different concentrations was based on the literature (Bahramparvar & Tehrani, 2011). The mixture was homogenized for 15 min, and the crude BE/BS‐based chocolate milk drinks were stored at 25 ± 1°C for 30 min. The drink samples were then transferred into clean tubes, and their turbidity was read at 600 nm using a turbidimeter HACH 2100N (Hach Company headquarters) following the method described by Desobgo (2012). Distilled water was used as the blank, and SDS was used as the positive control.

In addition to the turbidity of crude BE/BS‐based chocolate milk drinks, the in situ water absorption capacity and solubility index of crude BE/BS were assessed using the method described by Philips et al. (1988) with slight modifications. In the protocol, 100 mL of chocolate milk drinks were prepared with crude BE/BS at 0.5% (w/v). After 1 h of incubation at room temperature (25 ± 1°C), the drinks were centrifuged (3500×*g*, 10 min). The supernatants were discarded, and the bottoms were collected, weighed, dried at 105°C for 24 h, and weighed again. A chocolate milk drink free of crude BE/BS was used as the negative control, while SDS at 0.5% (w/v) was used as the positive control. The following formulas were used to calculate the water absorption capacity and solubility index of crude BE/BS:

$$\text{Water absorption capacity}=\frac{\left({\text{Mass}}_{\mathrm{wet}\;\text{bottom}}-{\text{Mass}}_{\text{dried bottom}}\right)}{\left({\text{Mass}}_{\text{dried bottom}}\right)}\times 100$$

$$\text{Solubility index}=\frac{\left({\text{Mass}}_{\mathrm{BE}/\mathrm{BS}}-{\text{Mass}}_{\text{dried bottom}}\right)}{\left({\text{Mass}}_{\mathrm{BE}/\mathrm{BS}}\right)}\times 100$$

### 2.8 Statistical analysis

All experiments were performed in triplicate, and the results were expressed as mean ± standard deviation. Analysis of variance and Duncan's multiple range test were performed to compare the means at *p* < .05. The statistical software Statgraphic Centurion XVI version 16.1.18 (StatPoint Technologies, Inc., 2012) was used.

## 3 RESULTS AND DISCUSSION

### 3.1 Screening of BE/BS production

**FIGURE 1:**
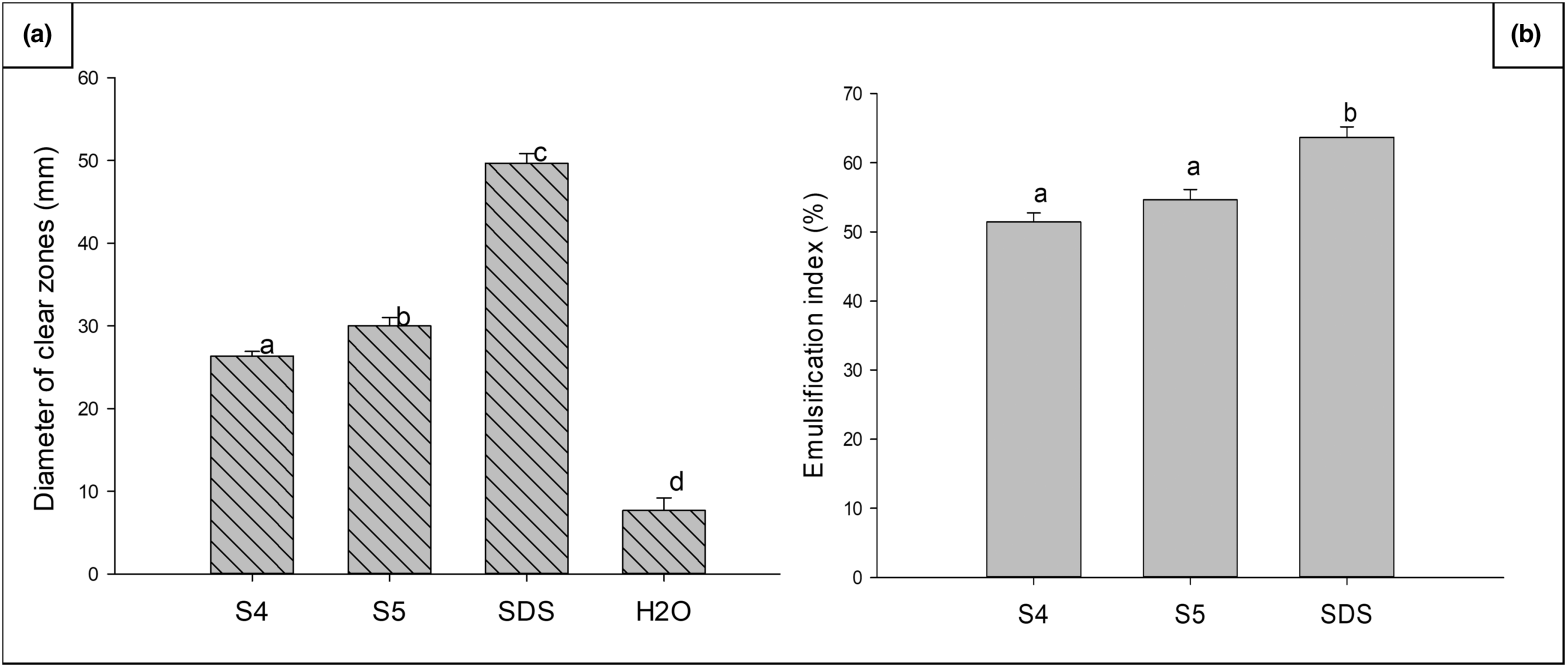
Zones of growth inhibition (mm) obtained from the oil spreading test (a) and emulsification index (b) of cell‐free supernatants of *Levilactobacillus brevis* S4 and *Lactiplantibacillus plantarum* S5. S4 = *L. brevis* S4; S5 = *L. plantarum* S5; SDS, sodium dodecyl sulfate. Different letters on histograms indicate a significant difference at *p* < .05.

In addition to the hemolytic activity and drop collapse tests performed in preliminary experiments to select the isolates, two more tests were used to confirm their ability to produce biosurfactants. They were the oil spreading test and the emulsifying activity. The oil spreading test, an indirect method that is more reliable in detecting low levels of biosurfactant production as determined by a tensiometer (Dong et al., 2019; Li et al., 2023; Yan et al., 2019), was used in this study to screen for the production of BE/BS by the isolates *L. brevis* S4 and *L. plantarum* S5. The two isolates showed positive results in the oil spreading test, with zones of growth inhibition of 26 and 30 mm for *L. brevis* S4 and *L. plantarum* S5, respectively (Figure 1a). These zones of growth inhibition were higher than 4.8 and 5.6 mm obtained by Yan et al. (2019) with biosurfactants from *Pediococcus acidilactici* 27,167 and *L. plantarum* 27,172, respectively, at a concentration of 50 mg/mL. This observation suggests the good production ability of our isolates as there is a positive correlation between the diameters of the clear zone from the oil spreading test and the concentration of biosurfactants (Dong et al., 2019; Li et al., 2023). However, surface tension measurement should be performed as an additional method to strengthen the biosurfactant production ability of the isolates, as Mukherjee et al. (2009) reported some limitations of the oil spreading test in detecting biosurfactants from certain microbial strains.

As depicted in Figure 1b, the supernatants from these isolates also showed emulsification indexes of 51.4% (*L. brevis* S4) and 54.6% (*L. plantarum* S5). This result demonstrates the presence of BE/BS in these supernatants, thus confirming the BE/BS‐producing ability of *L. brevis* S4 and *L. plantarum* S5 isolated from *pendidam*. Several studies have shown that the indigenous fermented milk called *pendidam* is a good source of lactobacilli that can produce biosurfactants (Fookao et al., 2022; Mbawala et al., 2013; Mouafo et al., 2022; Mouafo, Mbawala, et al., 2020; Mouafo, Mbawala, & Ndjouenkeu, 2018).

### 3.2 Production of bioemulsifiers/biosurfactants

BE/BS yields of 3.48 and 4.37 g/L were recorded from *L. brevis* S4 and *L. plantarum* S5, respectively. These yields were higher than those reported by Mbawala et al. (2013) with *Lactobacillus* spp. isolated from *pendidam* (0.40 to 0.54 g/L) and Mouafo, Mbawala, and Ndjouenkeu (2018) with *L. plantarum* (0.3 g/L), *L. delbrueckii* (0.51 g/L), and *L. cellobiosus* (0.49 g/L) isolated from *pendidam*. This difference might be ascribed to the polarity of the ethanol used as the extraction solvent in the present study. Indeed, ethanol extracts more compounds in terms of quantity than the ethyl acetate and methanol used by other authors, thus leading to the obtention of high BE/BS yields. A similar observation was reported by Radhakrishnan et al. (2010). However, the yields obtained in this study were lower than those reported by Fookao et al. (2022) for lactobacilli isolated from *pendidam* while using ethanol as the extraction solvent. This can be explained by the variability of BE/BS‐producing ability from one strain to another as a result of differences in metabolic activities (Tokumoto et al., 2009). Moreover, the chemical composition of biosurfactants from lactobacilli (a complex mixture of several components) could also explain the high yields obtained. Indeed, biosurfactants from lactobacilli are a mixture of polysaccharides, proteins, and phosphates that might have been precipitated by ethanol, leading to an increased yield. Given that the purification of biosurfactants leads to a significant reduction in their final yield (Mouafo, Pahane, et al., 2023; Salman & Alimer, 2014), the obtained BE/BS from *L. brevis* S4 and *L. plantarum* S5 should be qualified as crude extracts.

### 3.3 Partial characterization of bioemulsifiers/biosurfactants

#### 3.3.1 Surface and emulsifying activities

The surface activity of biosurfactants was assessed in this study using the oil spreading test, as this method was reported to be efficient in assessing the surface activity of biosurfactants (Brzozowski et al., 2011; Li et al., 2023; Yan et al., 2019). The crude BE/BS of the two isolates showed surface activities (22.47 ± 2.27 mm for *L. brevis* S4 and 26.74 ± 1.66 mm for *L. plantarum* S5). This surface activity could arise from the fact that the addition of BE/BS solution to the center of the oily film reduces the interfacial tension between the oily film and the distilled water present in the Petri dish, leading to the formation of a clear zone. The diameters of the clear zone obtained with crude BE/BS were not significantly different from those recorded with SDS (23.86 ± 2.02 mm). This result suggests the potential replacement of chemical surfactants in some industrial fields, such as the food industry and medicine.

The ability of crude BE/BS to form emulsions was assessed. Emulsification indexes of 51.17% ± 3.55% and 53.65% ± 4.14% were obtained with crude BE/BS from *L. brevis* S4 and *L. plantarum* S5, respectively. Although the emulsification indexes of BE/BS were not significantly different (*p* > .05) from those of the chemical surfactant SDS (52.43% ± 2.55%), they were lower than the values reported by Fookao et al. (2022) for BE/BS from lactobacilli isolated from *pendidam* (67.60% ± 3.30%, 66.23% ± 2.75%, 64.31% ± 0.96%, and 60.03% ± 3.33%). This could be ascribed to the differences in the chemical composition and structure of the biosurfactants. Structure‐related activities of biosurfactants have been highlighted in the literature (Durval et al., 2021; Liu et al., 2015; Mulligan et al., 2014).

However, emulsions formed by crude BE/BS from *L. brevis* S4 and *L. plantarum* S5 were stable after 72 h of incubation at room temperature, as no significant variations (*p* > .05) in emulsification indexes were noticed. This emulsion stabilizing ability of BE/BS could be explained by the fact that the adsorption of BE/BS at the interface between oil and air prevents the coalescence of droplets through electrostatic repulsion (Pinto et al., 2022; Rudy, 2011). Hence, the Ostwald ripening leading to the coalescence of droplets and an irreversible increase in the droplets' size (Karami et al., 2022) is reduced. The ability of BE/BS to control the clustering of globules and thus stabilize aerated systems was also reported in the literature (Lira et al., 2022; Santos et al., 2016). This result suggests that BE/BS is an alternative to chemical stabilizers used in the food industry, particularly in the preparation of chocolate milk drinks.

#### 3.3.2 Stability of the emulsifying and surface activities of BE/BS

*Stability to pH*: Although the emulsifying activity of crude BE/BS from *L. brevis* S4 slightly decreased at pH 6 and increased at pH 8, it remained stable from pH 4 to 8, as no significant difference (*p* > .05) was noticed (Table 1). At pH 10, a significant reduction (*p* < .05) in emulsifying activity was observed. This stability under acidic conditions could be attributed to electrostatic repulsion between drops that might be provoked by an increase in the negative charges of the carboxylic groups of BE/BS. Similar observations were made by Morais et al. (2017), Fookao et al. (2022) and Pinto et al. (2022). For the isolate *L. plantarum* S5, the emulsifying activity was stable at pH 6–10 (Table 1) but significantly decreased under acidic conditions (pH = 4). This stability under alkaline conditions can be attributed to the formation of stable micelles by fatty acids from BE/BS, as highlighted in the studies conducted by Mouafo, Mbawala, and Ndjouenkeu (2018) and Ribeiro et al. (2022). With regard to surface activity, the crude BE/BS of both lactobacilli strains were stable under alkaline conditions (Table 2). This can be explained by the precipitation of BE/BS under acidic conditions. Similar behaviors of biosurfactants under acidic conditions were reported by Mbawala et al. (2013), Gudiña et al. (2015), and Ghasemi et al. (2019).

**TABLE 1:** Effect of pH, NaCl, and heat treatment on the emulsification index of the bioemulsifiers/biosurfactants produced by *Levilactobacillus brevis* S4 and *Lactiplantibacillus plantarum* S5.

Parameters	*Levilactobacillus brevis* S4	*Lactiplantibacillus plantarum* S5	SDS
pH			
4	49.03 ± 3.51^bB^	40.66 ± 5.50^aA^	52.66 ± 3.21^aB^
6	48.56 ± 2.66^bA^	51.76 ± 4.05^bA^	49.32 ± 3.06^aA^
8	51.17 ± 3.55^bA^	53.65 ± 4.14^bA^	52.43 ± 2.55^aA^
10	44.66 ± 2.08^aA^	53.66 ± 3.78^bB^	58.48 ± 1.85^bC^
Temperature			
25°C/15 min	34.40 ± 1.15^aA^	43.50 ± 1.32^bB^	48.33 ± 1.52^bC^
50°C/15 min	36.66 ± 3.05^aA^	36.06 ± 2.64^aA^	44.66 ± 2.51^abB^
100°C/15 min	44.69 ± 5.03^bB^	33.50 ± 3.12^aA^	42.33 ± 3.05^aB^
121°C/15 min	37.05 ± 3.21^abA^	53.33 ± 3.51^cB^	47.68 ± 2.51^bB^
NaCl (g/100 mL)			
0	45.66 ± 2.51^bA^	47.33 ± 1.52^bA^	54.47 ± 1.78^aB^
25	52.33 ± 3.19^cB^	41.36 ± 1.15^aA^	63.04 ± 3.60^bC^
50	39.84 ± 2.08^aA^	55.66 ± 3.51^cB^	73.17 ± 3.60^cC^

*Note*: Values bearing similar superscript lowercase letters in each column and values bearing similar superscript capital letters in each raw are not significantly different at *p* < .05.

**TABLE 2:** Effect of pH, NaCl, and heat treatment on the surface activity of the bioemulsifiers/biosurfactants produced by *Levilactobacillus brevis* S4 and *Lactiplantibacillus plantarum* S5.

Parameters	*Levilactobacillus brevis* S4	*Lactiplantibacillus plantarum* S5	SDS
pH			
4	17.76 ± 1.77^aA^	18.50 ± 1.08^aA^	22.09 ± 1.54^aB^
6	22.47 ± 2.27^bA^	26.74 ± 1.66^cB^	23.86 ± 2.02^aA^
8	23.77 ± 2.09^bA^	25.66 ± 2.44^bcA^	22.57 ± 2.24^aA^
10	17.68 ± 1.80^aA^	22.32 ± 1.36^bB^	22.76 ± 1.58^aB^
Temperature			
25°C/15 min	23.66 ± 1.52^aA^	24.05 ± 0.57^aA^	24.09 ± 1.45^aA^
50°C/15 min	24.73 ± 2.51^aA^	25.33 ± 2.08^abA^	24.55 ± 1.96^aA^
100°C/15 min	29.08 ± 1.08^bA^	30.38 ± 1.52^cA^	28.07 ± 2.08^bA^
121°C/15 min	30.33 ± 2.08^bA^	28.64 ± 2.08^bcA^	31.66 ± 2.08^bA^
NaCl (g/100 mL)			
0	36.07 ± 4.58^aB^	35.08 ± 2.64^aB^	24.33 ± 1.52^aA^
25	38.66 ± 4.50^aA^	37.67 ± 1.15^abA^	35.33 ± 4.72^bA^
50	43.33 ± 2.08^aA^	40.33 ± 2.51^bA^	42.66 ± 2.51^cA^

*Note*: Values bearing similar superscript lowercase letters in each column and values bearing similar superscript capital letters in each raw are not significantly different at *p* < .05.

*Stability at different temperatures*: The surface activity of BE/BS from *L. brevis* S4 and *L. plantarum* S5 was stable after heat treatment at 25 and 50°C for 15 min, while it significantly increased after treatment at 100 and 121°C for 15 min (Table 2). Heat treatments at 25 and 50°C for 15 min and autoclaving at 121°C for 15 min did not show a significant (*p* < .05) effect on the emulsifying activity of BE/BS from *L. brevis* S4 (Table 1). A significant (*p* < .05) increase in emulsifying activity was observed after heat treatment at 100°C for 15 min. The emulsifying activity of BE/BS from *L. plantarum* S5 significantly (*p* < .05) decreased after heat treatment at 50 and 100°C for 15 min. However, autoclaving significantly (*p* < .05) increased the emulsifying activity (Table 1). Structural changes that might occur at high temperatures, leading to the formation of compounds with improved emulsifying and surface activities, could explain the stability of BE/BS observed in this study after autoclaving. The stability of biosurfactants under autoclaving conditions has been reported by Abdalsadiq et al. (2021), Fookao et al. (2022) and Gil et al. (2022).

##### Stability to salinity

The emulsifying activity of crude BE/BS from *L. brevis* S4 changes from 45.66% to 52.33% following treatment with NaCl 25%. However, it decreased significantly (*p* < .05) to 39.84% when treated with NaCl 50%. NaCl was also reported to significantly reduce the emulsifying activity of biosurfactants (Morais et al., 2017). The opposite phenomenon was observed with crude BE/BS from *L. plantarum* S5, as the emulsifying activity decreased from 47.33% to 41.36% when NaCl 15% was added and increased to 55.66% with NaCl 50%. A similar observation was reported by Fookao et al. (2022) using BE/BS from *Lactobacillus* sp. GM2B and might be due to the inhibition of proteins' relegation by phosphate and carboxylic groups present in the constitution of the biosurfactants. Indeed, biosurfactants from lactobacilli have been reported as a complex mixture containing sugars, lipids, proteins, and phosphates (Mouafo et al., 2022; Mouafo, Mbawala, & Ndjouenkeu, 2018; Rodrigues et al., 2006). Regarding the surface activity of crude BE/BS from both lactobacilli strains, an increase was noticed when the NaCl concentration increased. This result differs from those reported by Tharwat (2005), Mbawala et al. (2013), Gudiña et al. (2015), Bai and McClements (2016) and Oyedeji et al. (2022). They highlighted that at high NaCl concentrations, the charges of biosurfactants are hidden as the ionic strength of the medium increases, leading to a loss of surface activity. The results of this study suggest that further studies should be performed on these BE/BS in order to identify the mechanism by which surface activity increases in the presence of high NaCl concentrations.

The findings of this study regarding the stability of BE/BS at different temperatures, pH values, and NaCl concentrations suggest their potential application in several industrial fields where compounds with these behaviors are sought.

### 3.4 Antibacterial activity of bioemulsifiers/biosurfactants

Table 3 shows the inhibition diameters of crude BE/BS from *L. brevis* S4 and *L. plantarum* S5 against *E. coli* E1 and *S. aureus* S1. All the BE/BS were active, with the zone of growth inhibition ranging from 3.70 ± 0.22 to 5.70 ± 0.12 mm against *E. coli* E1 and from 3.83 ± 0.40 to 4.85 ± 0.42 mm against *S. aureus* S1. This antibacterial activity might be due to the amphiphilic nature of BE/BS, which enables their penetration into the phospholipidic membranes of bacteria, leading to alteration of their permeability, loss of their electrochemical gradient, and leakage of essential molecules (Makovitzki et al., 2006; Mbawala et al., 2013; Mouafo, Sokamte, et al., 2023; Sharma et al., 2023). Disruption of the bacterial cell wall and pores' formation following interaction with biosurfactants, as reported by Mouafo, Mbawala, and Ndjouenkeu (2018) and Mouafo, Sokamte, et al. (2023), could also explain the antimicrobial activity of BE/BS observed in this study. Although BE/BS from *L. brevis* S4 were more active than that from *L. plantarum* S5, they were less active than ampicillin and gentamicin. This might be ascribed to the purity of BE/BS, as it has been reported that the properties of biosurfactants increase with their level of purity (Mouafo, Baomog, et al., 2020; Mouafo, Pahane, et al., 2023).

**TABLE 3:** Antibacterial activity of crude bioemulsifiers/biosurfactants from *Levilactobacillus brevis* S4 and *Lactiplantibacillus plantarum* S5 expressed as inhibition zones (mm). Antibiotics gentamicin and ampicillin were used as controls.

Antibacterials	Inhibition diameters (mm)	
	*Escherichia coli* E1	*Staphylococcus aureus* S1
BE/BS		
*Levilactobacillus brevis* S4	5.70 ± 0.12^bB^	4.85 ± 0.42^bA^
*Lactiplantibacillus plantarum* S5	3.70 ± 0.22^aA^	3.83 ± 0.40^aA^
Antibiotics		
Ampicillin	7.10 ± 0.52^cA^	7.90 ± 0.11^cB^
Gentamicin	10.70 ± 0.32^dB^	9.15 ± 0.50^dA^

*Note*: Values bearing similar superscript lowercase letters in each column and values bearing similar superscript capital letters in each raw are not significantly different at *p* < .05.

Abbreviation: BE/BS, bioemulsifiers/biosurfactants.

After obtaining positive results from the qualitative test, the antibacterial activity of crude BE/BS was quantified by determining its MIC and MBC against the pathogens. Opposite to the qualitative test, BE/BS from *L. plantarum* S5 was active than that from *L. brevis* S4, as it showed the lowest MIC values (25 mg/mL) against both pathogens (Table 4). This observation suggests a difference in the chemical composition and structure of the two BE/BS. Variation in the antibacterial activity of biosurfactants according to the producing strain has been reported in the literature (Adnan et al., 2023; Liu et al., 2015; Mouafo, Baomog, et al., 2020; Sharma et al., 2023). These authors showed that the chemical structure of biosurfactants varies from one strain to another and that the activity of biosurfactants is directly related to their chemical structure. The MIC values obtained in this study are quite higher than 3.2 to 12.8 mg/mL found, respectively, against *S. aureus* and *E. coli* with biosurfactants from *L. paracasei* N2 (Mouafo, Mbawala, Tchougang, et al., 2018) and *L. casei* subsp. casei TM1B (Mouafo, Baomog, et al., 2020). However, they are in accordance with the MIC of 25 mg/mL reported by Sambanthamoorthy et al. (2014) for biosurfactants derived from *L. jensenii* 25,258 against *E. coli*, *S. aureus* UMS‐1, and methicillin‐resistant *S. aureus*. This could be explained by the difference in the chemical structure of these biosurfactants and the resistance patterns of the tested pathogens to antimicrobial compounds, which might vary from one bacteria to another.

**TABLE 4:** Minimum inhibitory concentration (MIC), minimum bactericidal concentration (MBC), and MBC/MIC ratio of bioemulsifiers/biosurfactants produced by *Levilactobacillus brevis* S4 and *Lactiplantibacillus plantarum* S5 against pathogens.

Pathogens	Parameters	*Levilactobacillus brevis* S4	*Lactiplantibacillus plantarum* S5
*Escherichia coli* E1	MIC	50	25
	MBC	100	75
	MBC/MIC	2	3
*Staphylococcus aureus* S1	MIC	50	25
	MBC	100	75
	MBC/MIC	2	3

The MBC of crude BE/BS was also assessed, and the results are presented in Table 4. BE/BS from *L. brevis* S4 and *L. plantarum* S5 showed MBC values of 75 and 100 mg/mL, respectively. For each BE/BS, no difference was observed between *E. coli* E1 and *S. aureus* S1, as the same MBC values were recorded for the two strains. According to the literature, when the MBC/MIC ratio of an antimicrobial compound is ≤4 against a pathogen, the compound is considered bactericidal against that pathogen (Teke et al., 2011). The MBC/MIC ratio was calculated in this study, and the results obtained (Table 4) showed that BE/BS produced by *L. brevis* S4 and *L. plantarum* S5 are both bactericidal against *E. coli* E1 and *S. aureus* S1. This bactericidal activity of BE/BS could be due to its ability to fix on bacterial cell walls and membranes through electrostatic interactions and alter these elements, which are vital for osmotic stability and the integrity of the bacterial cell (Inès & Dhouha, 2015). Sambanthamoorthy et al. (2014), Mouafo, Mbawala, Tchougang, et al. (2018), Mouafo, Mbawala, et al. (2020), and Mouafo, Mbawala, Tchougang, et al. (2018) also pointed out that bacterial cell walls and membranes are the most likely targets of biosurfactants. These results suggest the use of BE/BS as an alternative to chemical preservatives in the food industry or as an alternative to antibiotics in the medical field to reduce the phenomenon of drug multi‐resistance, which is now becoming a public health concern.

### 3.5 Application of BE/BS to the stabilization of cold chocolate milk drinks

As shown in Table 5, the turbidity of the cold chocolate milk drinks was directly proportional to the concentrations of BE/BS. Compared with the control without stabilizers, a significant (*p* < .05) increase was observed when BE/BS were added. According to the literature, stabilizers increase the turbidity of beverages and enhance their stability (Akkarachaneeyakorn & Tinrat, 2015). The increase in turbidity observed in this study could be explained by the gradual inhibition of the sedimentation of cocoa particles. Indeed, chocolate milk is a highly unstable drink. As time passes, the cocoa particles sediment, leading to the formation of a densely packed layer at the bottom. The consequence of this is a reduction in the turbidity of chocolate milk drinks (Aliakbarian et al., 2017; Sher et al., 2013; Vilela et al., 2018). The stabilizing properties of BE/BS may prevent that sedimentation, leading to the obtention of a turbid chocolate milk drink. Indeed, BE/BS can be absorbed at the interface of casein micelles' domains owing to their surface activity. The absorption of BE/BS onto casein micelles and its association with neighboring helices that adhere to these domains will stabilize the system and thus prevent coalescence and phase separation, as reported by Spagnuolo et al. (2005) in their studies with k‐carrageenan as a stabilizer of chocolate milk. The ability of surfactants to form hydrophilic colloidal dispersions leading to emulsion stability was reported by Vilela et al. (2018). Inhibition of particles settling following the incorporation of stabilizers in chocolate milk as the result of the formation of a colloidal network was reported by Tasneem et al. (2014). Thus, as the BE/BS concentrations in the mixture increase, the stability of the chocolate milk drink increases too, leading to a more turbid mixture compared with controls.

**TABLE 5:** Effect of bioemulsifiers/biosurfactants and sodium dodecyl sulfate concentrations on the turbidity of cold chocolate milk drinks.

Stabilizers' concentrations	*Levilactobacillus brevis* S4	*Lactiplantibacillus plantarum* S5	SDS
0	198.20 ± 0.78^aA^	198.20 ± 0.78^aA^	198.20 ± 0.78^aA^
0.2	256.58 ± 3.38^bA^	304.42 ± 2.64^bB^	315.55 ± 2.58^bC^
0.3	275.41 ± 4.03^cA^	320.19 ± 1.38^cB^	350.73 ± 2.08^cC^
0.5	355.30 ± 4.86^dA^	375.24 ± 3.02^dB^	415.17 ± 3.64^dC^

*Note*: Values bearing similar superscript lowercase letters in each column and values bearing similar superscript capital letters in each raw are not significantly different at *p* < .05.

Abbreviation: SDS, sodium dodecyl sulfate.

In the present study, the color of the drinks that became browner as the concentration of BE/BS increased could also explain the ability of these biomolecules to stabilize the brown cocoa particles in the mixture, leading to more turbid drinks. Indeed, the sedimentation of the brown particles of cocoa leads to a decrease in the pronounced brown color of the drink as time passes. The sedimentation of cocoa particles in drinks due to the absence of stabilizers was reported by Muhammad et al. (2021). The results of this study demonstrate the potential application of BE/BS as stabilizers in cold chocolate milk drinks and suggest their use as a substitute for chemical stabilizers. Moreover, the green nature of BE/BS associated with its numerous health benefits (antimicrobial, antioxidant, and antiadhesive activities), is an advantage of BE/BS compared with other stabilizers.

While considering the BE/BS, it appears that the one from *L. plantarum* S5 was significantly (*p* < .05) more active in stabilizing the chocolate milk drink, independent of the concentrations. This might arise from its high emulsification index of 53.65% compared with the one recorded with BE/BS from *L. brevis* S4 (51.17%). Although the stabilizing properties of BE/BS were lower compared with the chemical surfactant SDS, the green nature of BE/BS, associated with their lower toxicity, confers some advantages to these biomolecules.

To explain the behavior of BE/BS in cold chocolate milk drinks, their water absorption capacity and solubility index were measured. Crude BE/BS from *L. brevis* S4 scored a solubility index of 14.38% ± 0.67%, while the ones from *L. plantarum* S5 scored a solubility index of 16.43% ± 0.85% (Table 6). This can arise from the solvation of BE/BS in water through hydrophilic interactions. Indeed, BE/BS, due to their amphiphilic nature and surface activity, can be adsorbed at the interface between cocoa particles, milk components, and water molecules, resulting in their complete solubilization. A similar behavior was observed with the chemical surfactant SDS, for which a solubility index of 19.51 ± 0.95 was noticed. The values of the solubility indexes of BE/BS obtained in this study were higher than those of carrageenan recorded by Immoun (2015). According to Bahramparvar and Tehrani (2011) and Molet‐Rodríguez et al. (2018), who reported that a good stabilizer might have a high solubility index that will enable it to readily disperse in the mix, one can suggest BE/BS as a good stabilizer for the food industry.

**TABLE 6:** Water absorption capacity and solubility index of bioemulsifiers/biosurfactants and sodium dodecyl sulfate in cold chocolate milk drinks.

Stabilizers	Water absorption capacity (%)	Solubility index (%)
BE/BS from *Levilactobacillus brevis* S4	54.37 ± 0.57^a^	14.38 ± 0.67^a^
BE/BS from *Lactiplantibacillus plantarum* S5	56.13 ± 0.34^a^	16.43 ± 0.85^a^
SDS	63.43 ± 0.87^b^	19.51 ± 0.95^b^

*Note*: Values bearing different superscript lowercase letters in each column are significantly different at *p* < .05.

Abbreviations: BE/BS, bioemulsifiers/biosurfactants; SDS, sodium dodecyl sulfate.

The water absorption capacities of the crude BE/BS from *L. brevis* S4 and *L. plantarum* S5 were 54.37% ± 0.57% and 56.13% ± 0.34%, respectively. They were significantly (*p* < .05) lower than those recorded with SDS (63.43% ± 0.87%). The ability of BE/BS to absorb water through shear force and form a network with favorable features might explain the highest values of water absorption capacity obtained in this study. The consequences are an increase in the viscosity of the mixture and, thus, a rise in the turbidity of the chocolate milk drinks, as previously observed. A similar behavior of stabilizers was noticed by Ronkart et al. (2010) and Muhammad et al. (2021). The ability to absorb water molecules was reported by Abbasi and Rahimi (2006) as an important feature that a stabilizer must have because it contributes to increasing the resistance to flow and thus stabilizing the beverage. Eze et al. (2021) also highlighted that stabilizers bind water molecules, inhibit the separation of various ingredients, such as cocoa particles, and raise the viscosity of chocolate milk.

### 3.6 Principal component analysis

**FIGURE 2:**
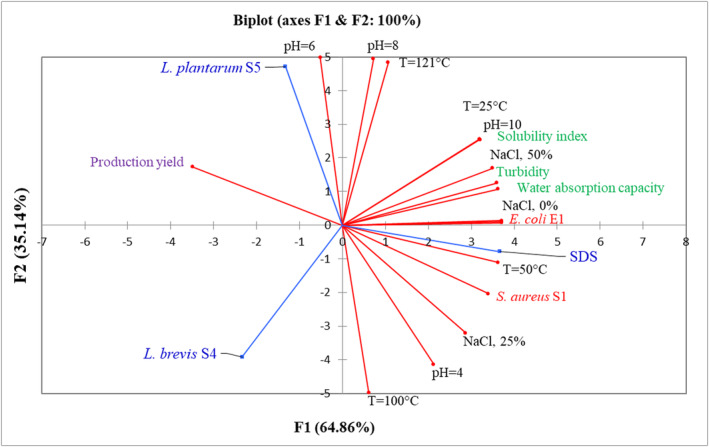
Principal components analysis of the antibacterial, functional, and stability properties of BE/BS produced by *Levilactobacillus brevis* S4 and *Lactiplantibacillus plantarum* S5. BE/BS, bioemulsifiers/biosurfactants; SDS, sodium dodecyl sulfate; T, temperature.

Using principal component analysis, the correlation circle of the different variables and the individual BE/BS and controls used in the study was drawn (Figure 2). The produced BE/BS samples, SDS positive control, *E. coli* E1 and *S. aureus* S1 strains, and resulting stabilizing properties have been projected in the single system. The principal component axes F1 and F2 expressed 100% variation among the different samples. It may be seen from Figure 2 that the F1 × F2 plot denoted a separation of BE/BS samples according to the bacterial strain. The first axis (F1, 64.86%) separated BE/BS from *L. brevis* S4 with BE/BS from *L. plantarum* S5, positioned on the left side of F1, and SDS positive control on the right. BE/BS samples were equally separated along the second axis (F2, 35.14%) according to the strain. In this respect, BE/BS from *L. plantarum* S5 were positioned on the upper side of the axis F2, while BE/BS from *L. brevis* S4 were found on the down side of this axis. It is observed that the water absorption capacity, the solubility index, the turbidity, the concentration of NaCl at 50%, and the temperature at 121°C are highly positively correlated with the BE/BS from *L. plantarum* S5, the strain *E. coli* E1, and the SDS control, while a negative correlation was observed with the BE/BS from *L. brevis* S4. As such, the results showed a significant positive correlation between the temperature of 100°C at pH 4, the NaCl concentration of 25%, the strain *S. aureus* S1, and BE/BS from *L. brevis* S4. This clearly indicates that all the physico‐chemical and microbiological parameters measured have an impact on the BS/BE samples produced. However, BE/BS from *L. plantarum* S5 have the highest production yield and exhibit higher stabilizing properties of cold chocolate milk drinks.

## 4 CONCLUSIONS

This study demonstrates the BE/BS‐producing ability of *Levilactobacillus brevis* S4 and *Lactiplantibacillus plantarum* S5 isolated from *pendidam* sold in the city of Ngaoundéré, Cameroon, and suggests *pendidam* as a good source of lactobacilli with high biosurfactant‐producing potential. The crude BE/BS from the two lactobacilli strains deserves emulsifying and surface activities that were stable under different temperatures, pH values, and salinities. They showed antibacterial activity against both Gram‐positive and Gram‐negative bacteria. When applied to cold chocolate milk drinks, the BE/BS from *L. brevis* S4 and *L. plantarum* S5 showed interesting solubility indexes and water absorption capacities, which led to the successful prevention of the sedimentation of cocoa particles. The results of this study highlight the potential use of BE/BS as stabilizers in the food industry and suggest that further studies should be performed to assess their effect on the sensorial and rheological properties of cold chocolate milk drinks.

## AUTHOR CONTRIBUTIONS

**Galvany Franck Yamagueu Tchakouani:** Investigation (equal); methodology (equal); writing – original draft (supporting); writing – review and editing (equal). **Hippolyte Tene Mouafo:** Data curation (equal); formal analysis (equal); validation (equal); visualization (equal); writing – original draft (lead); writing – review and editing (equal). **Richard Marcel Nguimbou:** Conceptualization (equal); data curation (equal); formal analysis (equal); software (lead); supervision (supporting); validation (equal); writing – review and editing (equal). **Nadège Donkeng Nganou:** Investigation (equal); methodology (equal); writing – original draft (supporting); writing – review and editing (equal). **Augustin Mbawala:** Conceptualization (equal); project administration (lead); resources (lead); supervision (lead); validation (equal); visualization (equal); writing – review and editing (equal).

## FUNDING INFORMATION

This research did not receive any specific grants from funding agencies in the public, commercial, or not‐for‐profit sectors.

## CONFLICT OF INTEREST STATEMENT

The authors declare no conflict of interest.

## ETHICS STATEMENT

The present study does not involve humans or animals.

## CONSENT TO PARTICIPATE

All the co‐authors are willing to participate in this study.

## CONSENT FOR PUBLICATION

All authors are willing for the publication of this manuscript.

## Data Availability

Upon request, the data used in this study are available from the corresponding author.
